# Successes and Challenges of HIV Mentoring in Malawi: The Mentee Perspective

**DOI:** 10.1371/journal.pone.0158258

**Published:** 2016-06-28

**Authors:** Emily Chien, Khumbo Phiri, Alan Schooley, Mackenzie Chivwala, John Hamilton, Risa M. Hoffman

**Affiliations:** 1 David Geffen School of Medicine, University of California Los Angeles, Los Angeles, CA, United States of America; 2 Partners in Hope Medical Center, EQUIP-Malawi, Lilongwe, Malawi; 3 Department of Medicine and Division of Infectious Diseases, David Geffen School of Medicine at University of California Los Angeles, Los Angeles, CA, United States of America; University of Washington, UNITED STATES

## Abstract

HIV clinical mentoring has been utilized for capacity building in Africa, but few formal program evaluations have explored mentee perspectives on these programs. EQUIP is a PEPFAR-USAID funded program in Malawi that has been providing HIV mentoring on clinical and health systems since 2010. We sought to understand the successes and challenges of EQUIP’s mentorship program. From June-September 2014 we performed semi-structured, in-depth interviews with EQUIP mentees who had received mentoring for ≥ 1 year. Interview questions focused on program successes and challenges and were performed in English, audio recorded, coded, and analyzed using inductive content analysis with ATLAS.ti v7. Fifty-two mentees from 32 health centers were interviewed. The majority of mentees were 18–40 years old (79%, N = 41), 69% (N = 36) were male, 50% (N = 26) were nurses, 29% (N = 15) medical assistants, and 21% (N = 11) clinical officers. All mentees felt that EQUIP mentorship was successful (100%, N = 52). The most common benefit reported was an increase in clinical knowledge allowing for initiation of antiretroviral therapy (33%, N = 17). One-third of mentees (N = 17) reported increased clinic efficiency and improved systems for patient care due to EQUIP’s systems mentoring including documentation, supply chain and support for minor construction at clinics. The most common challenge (52%, N = 27) was understaffing at facilities, with mentees having multiple responsibilities during mentorship visits resulting in impaired ability to focus on learning. Mentees also reported that medication stock-outs (42%, N = 22) created challenges for the mentoring process. EQUIP’s systems-based mentorship and infrastructure improvements allowed for an optimized environment for clinical training. Shortages of health workers at sites pose a challenge for mentoring programs because mentees are pulled from learning experiences to perform non-HIV-related clinic duties. Evaluations of existing mentoring models are needed to continue to improve mentoring strategies that result in sustainable benefits for mentees, facilities, and patients.

## Introduction

Approximately 70% of adults and children living with HIV (human immunodeficiency virus) reside in sub-Saharan Africa [1]. Malawi remains one of the hardest hit countries in the region, with adult prevalence of ~10.3% [2]. In 2014, an estimated 1.1 million people in Malawi were living with HIV, and HIV infection continues to be the leading cause of death [2]. Malawi is ranked one of the lowest resourced countries in sub-Saharan Africa with a gross national income per capita of $750. It has a population of 16.3 million [3], but only 0.343 nurses to 1,000 people [4], and a doctor to patient ratio of 1:110,195 HIV patients [5]. According to the World Health Organization (WHO), only two other countries have a lower density of physicians worldwide [4].

In 2005, the WHO recommended clinical mentoring to assist with the scale-up of HIV services and to address the need for “ongoing professional development to yield sustainable high-quality clinical care outcomes” [6]. Clinical mentoring provides support for health workers, improves patient care, and addresses the need for ongoing professional development to yield sustainable high-quality clinical outcomes [7, 8]. This strategy has been utilized for HIV capacity building in Malawi, South Africa, Rwanda, and Botswana [7, 9–11]. The limited body of knowledge available on benefits of clinical mentorship focuses on quantitative outcomes such as increased CD4 testing or improved patient documentation [10], but few publications have highlighted the experiences and perceptions of health worker mentees receiving mentorship.

Extending QUuality ImPprovement (EQUIP)-Malawi is a United States Agency for International Development/President’s Emergency Plan for AIDS Relief-funded HIV care and treatment program based at Partners in Hope (PIH) Medical Center in Lilongwe, Malawi, and has been providing HIV mentorship in Northern and Central Malawi since 2010. EQUIP mentors are experienced mid-level clinical providers (clinical officers and nurses) who provide mentorship to other mid-level providers (clinical officers, medical assistants, and nurses) in rural and urban Malawi hospitals and health centers. The hallmark of the EQUIP mentoring program is the integration of mentoring on clinical care and health systems topics allowing for building capacity of individuals and the larger systems in which they work. We sought to understand the successes and challenges of EQUIP’s mentorship program through qualitative in-depth interviews with mentees of the program. The goal of this study was to elucidate challenges, best practices, and lessons learned from EQUIP’s mentoring program to inform future mentorship efforts in Malawi and similar settings.

### EQUIP Mentorship Program

The EQUIP model involves mentoring in the setting of patient care. Mentors work side-by-side with mentees during outpatient adult and pediatric HIV visits, in antenatal clinics, maternity wards, and occasionally on inpatient wards. Mentoring also includes the use of didactic sessions and teaching on health systems utilization and systems improvements. Mentors are assigned mentees from health facilities within a District and travel to each EQUIP-supported site in the District to provide mentorship. The intensity of mentorship visits is divided into phases based on the needs at each site and involves intensive mentoring (weekly to bi-weekly) followed by maintenance mentoring (monthly visits). Sites that are doing well with clinical care and systems issues (based on mentee self-assessment, mentor assessment, and monitoring and evaluation data) “graduate” from EQUIP mentoring, but continue to receive quarterly visits to provide ongoing education and receive support for systems improvements.

Mentoring is provided across a range of topics including staging of HIV, initiation of antiretroviral therapy (ART), management of common ART toxicities, management of opportunistic infections (OIs), recognition of treatment failure, care related to the prevention of mother-to-child HIV transmission (PMTCT), early infant HIV diagnosis, viral load testing and interpretation, and care of HIV-exposed and infected infants. EQUIP mentors provide assistance in health system strengthening, including laboratory improvements (viral load testing, sample transport, results reporting), facility infrastructure (patient flow, efficiency, task shifting), procurement of supplies and medications, collecting and reporting of program indicators, and focused quality improvement for specific areas of weakness.

EQUIP mentees complete Ministry of Health self-assessment forms at baseline and at least quarterly throughout the mentoring relationship (S1 Fig). These forms capture areas of strengths and weaknesses, and are utilized for feedback sessions and to help mentees set and reach goals. The program also uses “dashboards”, which are color-coded tables that provide consolidated data on HIV health indicators reached by the facility (green = at or exceeding target; yellow = near target level; red = below target level) (S2 Fig). Mentors utilize dashboards to provide feedback to the providers on where the site is successful and where improvements are needed. The EQUIP model also utilizes the framework of building provider ‘networks’ to help support mentees. A network is comprised of a hospital and a group of surrounding health centers. Every 2 months mentees within the network meet with EQUIP leadership to review site-level challenges, national guideline changes, and to work on health systems challenges that can be addressed within the network, such as referral systems for specific medical conditions (tuberculosis, failure of first line ART) and systems that work across the network (sample transport, results reporting). Network meetings also link providers across sites and help build camaraderie and a community of HIV providers.

## Materials and Methods

This study was performed at health facilities in Northern and Central Malawi that are part of EQUIP-supported health centers and hospitals, including the following districts: Dowa, Lilongwe, Dedza, Mzimba, and Nkhata Bay. These districts included a total of 51 clinics with an average of 2 mentees at each site. Participants were selected to participate in a single, one-hour interview using convenience sampling based on the mentoring schedule. They were included if they were ≥ 18 years old and had participated as an EQUIP mentee for at least 1 year. Participants were approached about the study in person or by telephone. Ethical approval for the study was granted by the Malawi National Health Sciences Research Committee and by the UCLA Institutional Review Board, and committee representatives approved the use of oral consent for anonymous data collection.

A brief survey was administered to gather data on socio-demographics and clinical background, followed by a semi-structured, in-depth interview. The interviews were conducted in English by a university-trained Malawian researcher (author K.P.), with more than 3 years of experience performing qualitative interviews. Although the interviewer was EQUIP staff, she had no prior relationship or interaction with site mentees. Interviews were conducted in a private space at the clinic or hospital with only the interviewer and participant present. Interview questions focused on the mentorship experience, mentor/mentee relationship, and overall impressions with an emphasis on successes, challenges, and areas for improvement (Table 1). The instrument was pilot tested with ten individuals not participating in the study and was adapted based on feedback from this group.

**Table 1:** Study Population Characteristics Stratified by Sex.

	Men(*N* = 36)	Women(*N* = 16)	^a^p-value	Total(*N* = 52)
**Age category (years) % (N)**				
**18–25**	0% (0)	25% (4)	<0.001	8% (4)
**26–29**	19% (7)	19% (3)	1.00	19% (10)
**30–40**	67% (24)	19% (3)	0.002	52% (27)
**41–50**	11% (4)	19% (3)	0.66	14% (7)
**> 50**	3% (1)	13% (3)	0.08	8% (4)
**Type of Health Care Worker % (N)**				
**Clinical Officer**	31% (11)	0% (0)	0.01	21% (11)
**Medical Assistant**	33% (12)	19% (3)	0.34	29% (15)
**Nurse**	36% (13)	81% (13)	0.006	50.0% (26)
**Region % (N)**				
**Northern**	39% (14)	56% (9)	0.36	44% (23)
**Central**	61% (22)	44% (7)	0.36	56% (29)

^a^p-value calculated for comparisons of men versus women using Fisher’s exact test.

Quantitative socio-demographic data were analyzed using STATA (StataCorp version 12, College Station, TX) to generate summary statistics. Categorical variables are presented using proportions (%, N) and continuous variables are presented as medians and interquartile ranges (IQR). Fisher’s exact test was used for comparisons of socio-demographic variables between men and women. Interviews were performed in English, digitally audio recorded, coded, and analyzed with ATLAS.ti version 7 (Berlin, Germany). Inductive content analysis was used to identify themes from the interviews [12]. A code list was derived based on common themes [13]. Proportions of themes were calculated based on number of respondents mentioning the theme divided by total number of respondents. Themes that occurred more than once in a single interview were counted once.

## Results

Fifty-two mentees were approached about the study and all consented to participate. These individuals were from 32 EQUIP-supported health centers and hospitals in Central and Northern Malawi. The sample represents 43% of mentees and 63% of sites supported by EQUIP. The majority of mentees were between the ages of 18 and 40 (79%, N = 41) and 69% (N = 36) were male. Of the 52 mentees, 50% (N = 26) were nurses, 29% (N = 15) medical assistants, and 21% (N = 11) clinical officers (individuals who complete three years of school and a one-year internship and receive a Diploma in Clinical Medicine). Forty-four percent of mentees (N = 23) were from Northern Malawi EQUIP-supported health facilities and 56% (N = 29) were from Central Malawi. Data stratified by sex are included in Table 1. Women were more likely to be between the ages of 18 and 25 (25% versus 0%, p < 0.001) and men were more likely to be between the ages of 30 and 40 (67% v. 19%, p = 0.002). Men were more likely to be clinical officers (31% versus 0%, p = 0.01), while women were more likely to be nurses (81% v. 36%, p = 0.006). The median number of years that mentees worked at their current facility was 4 (IQR 2–7 years). Mentees had a median of 1 year (IQR 0–3 years) of HIV/AIDS clinical experience prior to EQUIP mentorship and had a median length of 2 years (IQR 1–2 years) of EQUIP mentorship. The majority of mentees (58%, N = 30) did not receive mentorship from other programs during EQUIP mentorship at the time the interview was performed. Amongst those that received outside mentorship, most encounters were Ministry of Health supportive supervision visits, which are comprised of a quarterly visit from a government team to review documentation required for national program monitoring and evaluation.

### Successes and Facilitators of EQUIP Mentoring

When asked whether EQUIP mentoring was successful, all mentees answered affirmatively (100%, N = 52). One provider stated:

*“After mentorship … we are able to run the clinic without mentors*, *without referring to other people*, *without waiting for people from [EQUIP] …*. *we are able to treat the patient … and now the number of patients has grown that are coming to clinic*.*”–*KAS056 (clinical officer, Central Malawi)

Mentees mentioned that their previous ART training was too limited to provide sufficient experience in the various different types of HIV case presentations at their clinics. They felt that the mentorship program provided practical experiences in managing HIV patients across a range of ages and clinical scenarios, including pregnancy. A nurse mentee stated:

*“I think my experience has been very good because the mentorship helped me to learn more [on] the care of those who are HIV-positive or pregnant women …*. *I have seen great improvement because we [are] not be able to learn everything on [our] own*, *you need someone to help you when you are not okay*. . . . *I think these people have really helped us in the mentoring process*.*”*–MBI017 (nurse, Central Malawi)

A medical assistant from one of the central sites for EQUIP stated:

*“Yes I think it has been successful … people are being helped*. *Without EQUIP*, *I think I would be somewhere in doubt … the Ministry of Health training is a 3-day training … [with] EQUIP*, *I am still learning*, *so I am proud*.*”–*DIA026 (medical assistant, Central Malawi)

The most common benefit from EQUIP mentorship reported by mentees (33%, N = 17) was the increase in clinical knowledge that allowed them to initiate ART for HIV-infected patients at their sites.

*“We have been able to initiate more clients on ART than any other years*. *We are able to do CD4*, *accept patients*…*and do staging*.*”*–NAT025 (nurse, Central Malawi)

*“Because of the partnership with mentors*, *we were able to initiate [this patient] on second line ART …*. *The patient has got a baby boy now and she is doing very well now*.*”*–NKH031 (clinical officer, Central Malawi)

*“The mentors are just so quite good because I was really new*. *I did not know anything about the ART clinic so when EQUIP came in*, *I was much helped with them …*. *I got a lot of information because of them*.*”*–MAD008 (nurse, Central Malawi)

*“I can say I have been enjoying the mentorship …*. *I would not even know the concept of HIV/AIDS …*. *EQUIP started mentoring us and I knew HIV/AIDS before I went to initial training conducted by Ministry of Health …I was proud of that*.*”*–DIA026 (medical assistant, Central Malawi)

One-third of mentees (N = 17) reported that, as a result of EQUIP mentorship, they had received certificates from the Ministry of Health. Of these individuals, 47.1% (N = 8) felt that a key component allowing achievement of the certificate was EQUIP-supported improvements in basic equipment and/or minor construction that allowed for improved quality of care, 47.1% (N = 8) attributed the success to self-assessment forms that helped them to identify gaps in their clinical knowledge, and 35.3% (N = 6) reported achieving certifications because EQUIP mentors taught them about the Malawi National HIV/AIDS Guidelines.

*“Yes I feel very comfortable because last time we were not given a certificate for almost two years but this year we are going to receive a certificate … we are doing well*. *They [Ministry of Health officers] are saying there is no need for you to have mentors here*, *you can mentor somebody yourself here*.*”*–MAD010 (nurse, Central Malawi)

*“The most important part which I like most is*, *when I started in the ART clinic*, *I had only knowledge from [Ministry of Health] training but I had no experience … but when [I] started being mentored by the EQUIP guys*, *I have learned a lot …*. *At first we were around 50% [on quarterly supervision] but since these guys came*, *we have been doing very*, *very fine*. *We have been 100%*. *So now I am very comfortable*.*”*–KAY011 (nurse, Central Malawi)

*“I was training with EQUIP to be an ART provider* … *they mentored me to know more about this infection*, *and the supervisors from Ministry of Health were able to evaluate me that I am doing better*, *that’s why they gave me a certificate … everything in this certificate belongs to EQUIP*. *The knowledge I am speaking here goes to EQUIP because they are the ones who imparted this knowledge to me*.*”*- MTW045 (nurse, Northern Malawi)

One-third of mentees (N = 17) reported that patient encounters greatly benefitted from EQUIP’s investment in basic supplies, equipment, and minor construction at clinics.

*"Now we have got more space*, *more room to conduct the clinics*. *We have been provided with furniture where we can examine patients*. *We have been provided with equipment to provide proper care like height*, *weight scales*…*the number of stock-outs of test kits have greatly been reduced*.*—*MAD018 (clinical officer, Central Malawi)

*“At first we were giving our ARVs in one of those rooms there*, *so when these EQUIP people came they said ‘No*, *this is not comfortable for your patients*, *please change*. *Get some other rooms where you will be giving your ARVs*.*’ When we looked at it*, *we said true … we can’t be giving drugs here*, *we can’t be seeing patients here*. *There was no privacy*, *no confidentiality*, *things like that*. *So because of them we changed and came here … now we have privacy*, *confidentiality and there is space*.*”–*CHI050 (nurse, Northern Malawi)

Several mentees (10%, (N = 5) specifically cited the importance of EQUIP’s role in helping address drug stock-outs:

*"If there is a stock-out*, *they've been very helpful*. *Even calling the HIV/AIDS unit*, *even in transportation of the drugs … [they] take some drugs that are out of stock and bring them to us*.*”*—MAD018 (clinical officer, Central Malawi)

*"Very much successful because they helped us not only in mentoring us but also … whenever we have stock-outs*, *they are the ones who try to collect the drugs for us*.*”*—NTH021 (clinical officer, Northern Malawi)

Fifty-six percent (N = 29) of mentees stated that quarterly dashboards were helpful in identifying areas for improvement and recognizing program successes.

*“Yeah*, *because they [dashboards] give percentages … if you have got 100%*, *you see we have done well*. *But if you have scored less than 50% you see*, *ah*, *we are a little far*. *We need to improve and [the dashboard] really helped to change some areas*.*”—*MBI017 (nurse, Central Malawi)

*“Most of the time we would discuss [quarterly dashboards] during the network meetings and you could see how your facility is fairing compared to other facilities [this] would give you a chance to see the problem and why you were not achieving certain targets*, *why certain percentages are low in certain areas …*. *We were given a chance to sit down and discuss how we can improve in certain areas … it really did help*.*–*MAD018 (clinical officer, Central Malawi)

*“I was able to see the areas where I am not doing better*. *They were indicating red … you already know that you are doing bad*. *I think it makes you to do something better so that the red does not appear on your dashboard*.*”–*MTW045 (nurse, Northern Malawi)

In addition to dashboards, self-assessment tools were also highly rated by mentees. Sixty-two percent (N = 32) reported that self-assessment helped identify gaps in knowledge and serve as a guide to assist the mentees in building the skills necessary to see patients independently.

*“They [self-assessment tools] are really helpful because you now assess yourself with those questions … it’s where you can see where you need help and where you learn you are confident*. *When you are confident you know that when a patient comes here you can do this and that … when you evaluate yourself*, *you self-assess then the mentors know where to start with you*. *They know where you are deficient*.*”*–BOW006 (medical assistant, Central Malawi)

*“Those forms comprised some topics which we didn’t know*, *so they were guiding us … I had to fill [out the self-assessment form] so that when these mentors come*, *they help me and teach me how to do some things … [the] forms were helpful to me*.*”*–CHI013 (medical assistant, Central Malawi)

Network meetings were another facilitator of success mentioned by mentees (21%, N = 11). Meetings were considered an important opportunity to share lessons learned, to connect with colleagues and friends providing HIV care at other sites in the District, and as a space to find support and encouragement.

*“I think if we could continue the [network] meetings because we meet with several people from several facilities so we share and you find that I am also behind and this is what my friend is doing*, *let me do the same as [my friend] is doing*. *So it was encouraging each other*. . . . *I can say they were just very fruitful meetings to me*.*”–*DIA026 (medical assistant, Central Malawi)

*“Those [network] meetings*. *I wish they would be done every 3 months because … we learn from other peoples’ mistakes …*. *Whenever we go to those meetings … we discuss the dashboards or sometimes some errors that are made at our facilities …*. *I think during those meetings we learn a lot from our friends*.*”–*NTH 021 (clinical officer, Northern Malawi)

Network meetings also provided a setting where problems could be discussed and solutions addressed:

*For example*, *the percentage of women tested for HIV in antenatal*, *you will find stock-outs of test kits …*. *We made a solution*, *we should reserve some test kits for the antenatal mothers so the priority of test kits was given to antenatal mothers … one of those solutions came out because of the network meetings*. *We would come up with solutions and implement them*. *And consider that things really improved greatly*.*”–*MAD018 (clinical officer, Central Malawi)

Table 2 summarizes the success and facilitators as reported by mentees.

**Table 2:** Success and Facilitators of EQUIP Mentorship (N = 52).

	% (N)
Successes	
Improved clinical knowledge	33% (17)
Now receiving Ministry of Health certificates	33% (17)
Infrastructure support	33% (17)
Facilitators	
Use of self-assessment forms	62% (32)
Use of quarterly dashboards	56% (29)
Network meetings	21% (11)

### Challenges and Barriers to Mentoring

The most common challenge reported by mentees (52%, N = 27) was understaffing at health facilities, requiring the mentees to have multiple responsibilities at their site during mentorship visits.

*“There was a particular time when I was alone … I had to work in the labor ward*, *antenatal clinic and the outpatient department …*. *During Wednesdays*, *I had to leave some clients in antenatal and the outpatient department and we were concentrating much on ART clinics*. *But you know we cannot leave a client in labor ward and then [conduct] some sessions at ART … it was a challenge because I was leaving the mentors [to] attend the client in labor ward … [the timing] was a challenge*.*”—*CHI013 (medical assistant, Central Malawi)

*“Sometime I just left the job to the mentors because I am alone*, *I am supposed to cover for OPD [outpatient department] as well as maternity as well as ART*. *So sometimes because of the workload*, *instead of me being mentored*, *I just leave the job to them*. *So that’s the only challenge which I am having now*.*”–*KAY011 (nurse, Central Malawi)

*“It’s easy to make a lot of mistakes because you lose concentration because of the work load*.*”–*MAT029 (medical assistant, Central Malawi)

*“Interruptions [are a problem]*. *We are doing clinic here*, *then counseling the other patient there*. *I was forced to leave the clinic to attend to the patient there*.*”*–KAM048 (medical assistant, Northern Malawi)

Forty-six percent of mentees (N = 24) described lack of space and/or privacy as an important barrier to mentoring and patient care in ART clinics.

*“The first issue is space because we have been conducting our clinic in here yet it is [also the] labor ward …*. *That is the biggest challenge … we don’t have a specific space to provide ART*.*–*KAY011 (nurse, Central Malawi)

*“To us the main challenge will be the infrastructure thing because the room we are using for ART is too small and you have to double for OPD [outpatient] like consultation room … we would have preferred to have a separate room especially with ART because there are a lot of issues like you need to keep your drugs there*. *You need to keep the mastercards there …*. *I will have to look for keys on this place which I don’t have and then get the drugs*. *Maybe I just give the client the drugs … then I would have to search for the mastercards*. *But if there is one room*, *you have everything in that room*, *it would not be difficult*.*”*–MBI015 (medical assistant, Central Malawi)

*“There were issues of infrastructure*. *Medical confidentiality was a challenge to meet because the place was just too small and … after we have started on services … we had a lot of clients enrolled in a short period of time [in the same space]*. *Some were even waiting outside to be attended to*. *So basically space was not adequate*.*”–*MAP057 (clinical officer, Northern Malawi)

Lack of furniture for clinics was another problem that was cited as a challenge to mentorship.

*“Our rooms [are] very small because we can have like four clients or two clients with their relatives … and the main thing [is] the space*. *There’s not enough chairs and the others are sitting on the floor*. *It’s not good for the client to be on the floor like that [and] to be treated like that*.*”*- MBI017 (nurse, Central Malawi)

*“The setup of the facility*. *Our rooms are not good so usually the mentor will have to stand or sit on the chair*. *The patient will sit and the mentor has to stand so I don’t think that’s comfortable*.*”*–BOW006 (medical assistant, Central Malawi)

Forty-two percent (N = 22) of mentees mentioned that stock-out of drugs at their facilities hindered mentorship sessions. Inadequate sample transport (15%, N = 8) also limited the opportunities to discuss labs during patient encounters. Table 3 summarizes the challenges as described by mentees.

**Table 3:** Challenges of EQUIP Mentorship (N = 52).

	% (N)
Understaffed/mentee multiple responsibilities	52% (27)
Not enough space/privacy/lack of furniture	46% (24)
Stock-outs of medications and/or supplies	42% (22)
Lack of sample transport	15% (8)

### Perspectives on Mentoring Style and Opportunities for Program Strengthening

The most common mentorship techniques were observation of mentees with corrections during the encounter (83%, N = 43) or after the encounter (33%, N = 17) and demonstration by the mentor with mentee observing (35%, N = 18). Approximately half of mentees preferred mentor observation of mentee with corrections and feedback during the encounter (48%, N = 25) while the other half preferred to have feedback after patient encounters (42%, N = 22).

*“[If] there was something that I have missed*, *they would pick it apart … and when the patient goes*, *they say okay you missed this point*, *once the other comes you do it like this*, *and when the next patient comes*, *you do better*.*”–*KASE005 (nurse, Central Malawi)

*“[The mentors] see how I do [the work]*, *any gaps [they] come and help me*, *at the end of the session*, *we sit down and discuss*, *so I think this style we are currently using is just fantastic*.*”–*KASE003 (nurse, Central Malawi)

*“They combine demonstration*, *lectures … sometimes we sit and discuss [a] topic…*. *I can say there is no problem … maybe sometimes there is an assignment … and next time when [the mentors] come*, *we will discuss*.*”–*MPA024 (nurse, Northern Malawi)

A minority of mentees (27%, N = 14) liked having the mentors demonstrate skills to them while the remainder (73%, N = 38) felt that this strategy was not ideal for learning. One mentee stated:

*“In some other cases*, *it’s like the mentor is doing the work and the mentee is observing*, *which is not good …*. *It has to be the mentee doing the work … and then the mentor corrects you…*.*”–*BOW006 (medical assistant, Central Malawi)

When asked to describe the ideal characteristics of a mentor, mentees described the following: friendly (44.2%, N = 23), knowledgeable (36.5%, N = 19), open (23.1%, N = 12), approachable (19.2%, N = 10), and “not harsh” (19.2%, N = 10). Preferred styles of mentorship are summarized in Table 4.

**Table 4:** Preferred Style of Mentorship (N = 52).^a^

	% (N)
Mentor observes and corrects during encounter	48% (25)
Mentor observes and corrects after the encounter	42% (22)
Mentor and mentee work together and/or discuss case during patient encounter	42% (22)
Demonstration of skills or clinical care	27% (14)
Presentation of topics before patient encounters	19% (10)
Follow-up lecture or discussion after patient encounters	13% (7)
Group discussions with other mentees	6% (3)

^a^ Individuals could list more than one preferred format for mentoring.

When asked about areas for program strengthening, the majority of mentees stated that they would like to have more visits from mentors (34.6%, N = 18), more space and privacy to be mentored and see patients (32.7%, N = 17), more frequent network meetings (19.2%, N = 10), and reference materials and learning aids to use between mentorship sessions (19.2%, N = 10). In regard to reference materials, one mentee reported:

*“Yes*, *even though I have mastered a lot of things … if there are some booklets [for] when I forget*. *[The mentors] will not be here all the time so when they’re not here … I will not remember everything … we can have those materials from training*.*”–*BOW006 (medical assistant, Central Malawi)

Additional themes included the need for more investment in supplies and equipment (15%, N = 8), increased focus on self-evaluation (13%, N = 7), and more training on inpatient medicine (12%, N = 6).

## Discussion

Clinical mentorship is a recommended tool to aid in the rapid scale-up of HIV services in resource-constrained settings [6]. Consistent with findings from previous mentorship studies, we found that clinical mentorship added to provider knowledge and confidence [14, 15], increased comfort in medication management [10], and supported rapid scale-up of HIV services through improved ability to initiate ART [8].

EQUIP has faced systems and infrastructure challenges shared by other health capacity-building programs including staffing limitations [16, 17], inadequate knowledge transfer processes [7], physical space limitations [18, 19], intermittent drug stock-outs [19, 20], and limited laboratory and pharmacy capacity [21]. Our program initially planned to focus mentoring on clinical topics related to HIV care; however, in the early assessment phase of the program, we learned of the barriers faced by clinics and felt that clinical mentoring with simultaneous systems strengthening and mentoring in systems improvements would provide the best chance for sustainable program improvements. Our qualitative data demonstrate that the use of an integrated clinical and systems-based mentorship approach is valued by mentees and participants in the study reported an increase in the appropriate utilization of the systems of care around HIV. Many of the key successes described by mentors resulted from improved systems. EQUIP mentorship has been successful because clinical topics have been supplemented with topics around the systems of care, such as privacy management, patient documentation, infrastructure investments, and strengthening of laboratory systems. This comprehensive approach to HIV mentoring provides support for sustainable improvements in the continuum of HIV care.

The lack of basic facility infrastructure (space, furniture, basic supplies and equipment) was a major barrier reported by mentees. This is a sentiment shared by other programs focused on HIV health delivery [7, 16, 22]. EQUIP was able to provide clinical sites with scales, chairs for patients, desks for providers, cabinets for patient files, and to perform minor construction to improve clinic flow, efficiency, and patient privacy. These changes allowed mentors to focus on teaching rather than creating workarounds to overcome infrastructure challenges. These infrastructure improvements are also low cost, rapid solutions that can add to the positive experience of mentees, productivity of mentorship, and improve the quality of health services. At least one study has shown that infrastructure improvements can increase uptake of services in HIV patients [18]. The ability to provide infrastructure improvements may be limited by available funding and data showing the beneficial outcomes of these investments are needed to advocate for program spending.

A unique component of EQUIP was the focus on strategies to build site-level ownership of HIV programs through the use of networks and dashboards, both highly rated by mentees. Network meetings create a community of HIV providers and provide a forum to discuss difficult clinical cases, problem solve around health systems issues, and develop standards for systems that function across sites such as patient referrals and laboratory services. EQUIP leadership continue to participate in network meetings after sites graduate from the program allowing a sustained connection with these graduated sites and providing opportunities for continuing medical education and support for health system challenges. Networks have become an important component of the sustainability of improvements that have resulted from EQUIP mentoring and may be a strategy for other mentoring programs in similar settings.

Dashboards are a second strategy for building site ownership and sustainability. Site personnel can be trained to create dashboards from indicator data. This is an inexpensive tool that allows for real time feedback about components of the clinical program. Mentees had positive evaluations of dashboards and expressed pride in seeing improvements. Dashboards can also be shared at network meetings (across sites) to understand similarities and differences in health system challenges and to share strategies for overcoming these challenges.

A limitation of the EQUIP mentoring model has been high turnover of clinical providers at sites. This is a well-documented issue in resource-constrained settings, poses a major problem for long-term institutional knowledge, and contributes to strain on facilities already faced with limited resources [23]. There were several instances in which EQUIP mentors needed to return to a previously “graduated” site to re-initiate mentorship due to loss of the single HIV provider previously mentored through the program. As a result of this challenge, EQUIP is considering a new model of mentoring in which providers from more remote sites are brought to a high volume HIV clinic that serves as a Center of Excellence for training while an EQUIP clinician “fills in” at the remote clinic site. This model decreases the investment of time and infrastructure (e.g., vehicles, fuel, accommodation, etc.) in sites where a single provider is being mentored and lessens the loss if the individual leaves the site. This strategy also allows the mentee to focus entirely on HIV training without the burden of additional clinical duties that are often present at their site. A challenge of this approach is finding an appropriately trained EQUIP clinician (who may be required to cover maternity and surgery wards in addition to HIV clinics). This approach also introduces the mentee to a set of resources at the Center of Excellence that may not be available in a more remote rural site (expanded pharmacy options, radiology services, and laboratory resources, among others). On balance, multiple mentoring and training strategies will be required to achieve the goals of building capacity for HIV care in Malawi. EQUIP is working towards utilizing both the central training site (Center of Excellence) and the mobile training unit, with decisions about the approach based on the needs, resources, and “fit” for particular sites and individuals.

### Study Limitations

This study was performed as a program evaluation to gain information on the challenges and successes of the EQUIP mentoring model and therefore we are unable to compare our mentoring approach to that of other programs. We used convenience sampling and were not able to interview every individual mentored by EQUIP or to include every site in our program. As such, data obtained does not represent the views of all mentees and sites, and our data may not be generalizable to all HIV care and treatment programs in resource-limited settings. EQUIP sites may receive mentorship from other non-governmental organizations and from the Ministry of Health. While efforts were made to understand the specific influence of EQUIP mentoring separate from external mentoring, we acknowledge that we cannot completely control for the possible impact of other mentoring on mentee perspectives. Our program provided transport to network meetings and we understand that this may not be feasible for all programs interested in supporting networks. Our study focuses on the qualitative perspectives of the mentees but does not include quantitative outcome measurements such as improvement in clinical knowledge, physical exam skills, and appropriate utilization of diagnostic tests and treatment guidelines. We utilized an EQUIP staff member to conduct the participant interviews. We tried to minimize the risk of bias by collecting data anonymously and by selecting a staff member who was not in a position of authority over mentors and did not work directly with mentors or make regular site visits to participant facilities. However, we cannot exclude the possibility that EQUIP mentors withheld information because of concerns about confidentiality.

## Conclusions

EQUIP’s integrated approach to HIV mentorship, including both clinical and systems-based training, was viewed as a successful model by mentees. The addition of systems-based mentorship and infrastructure improvements allowed for an optimized environment for clinical training. Site network meetings and dashboards have been important tools towards the development of site-ownership and sustainability of quality HIV care. Future studies of mentoring should include the use of objective measurements of mentoring, such as individuals’ knowledge and skills gained and improvements in rates of appropriate utilization of diagnostics and treatment guidelines. EQUIP’s clinical and systems-based mentoring model is a promising strategy to expand HIV care and improve the quality of health services for people living with HIV in Malawi and similar settings.

## Supporting Information

S1 Fig MOH Self-Assessment Form. (PDF)

S2 Fig Quarterly Dashboard Report. (PDF)
