# Supplementary material for: Successes and Challenges of HIV Mentoring in Malawi: The Mentee Perspective
Source: PLoS One. 2016 Jun 28;11(6):e0158258. doi: 10.1371/journal.pone.0158258 (PMC4924818; doi:10.1371/journal.pone.0158258)
Supplement: S1 Fig — (PDF) [file pone.0158258.s001.pdf]

Version Date: 10/05/2013

Mentee Checklist

Date of visit: DD / MM / YYYY

Please Circle: Initial or Quarterly

**Mentee Name:**		**Mentee position:**	
**Mentor Name:**		**Clinic name:**	

INSTRUCTIONS

On a quarterly basis, the mentee fills in the 'Mentee self-assessment' section in full before the mentor completes their section: the mentee considers each item and marks whether they feel confident to perform it independently, whether they need more support, or N/A ("not applicable") if it is not their responsibility.

The mentor fills in the 'Mentor assessment' column scoring the mentee from 1-3 (**1: Needs intensive support; 2: Needs some support; 3: Competent** - little or no support required) on each item or marking N/A if the item is not the mentee's responsibility.

When the assessment is complete, the mentor totals the scores for each section and counts the number of N/As marked (only in the mentor section, the mentee self-assessment is not used for scoring). The scores are then written in the boxes below the table using the formula written below to calculate the percentage score for the section. This percentage is then translated as either: red (0-49%), orange (50-79%) or green (80-100%):

EXAMPLE:

Totals: a 15 b 3

Calculate total score: (100 x a 15) ÷ (30 – b 3) = 56 %

Based on %, tick: [ ] RED (0-49%) [✓] ORANGE (50-79%) [ ] GREEN (80-100%)

OVERALL SCORE

Instructions

Transfer the % scores from each section, add them up and divide by 4 to get the overall % score, which should be translated into the mentoring phase.

Section:		% score for each section:	
A. Pre-ART			%
B. ART	+		%
C. PMTCT/ paediatrics	+		%
D. Other topics	+		%
Overall score:	=		÷ 4 = ______ %

Based on overall %, tick: **[ ] RED (0-49%)** **[ ] ORANGE (50-79%)** **[ ] GREEN (80-100%)**

Mentee Self-Assessment and Mentor Assessment

• Mentee Self-Assessment Column = Completed by Mentee
• Mentor Assessment Column = Completed by Mentor

Mentor scoring:
1: Needs intensive support
2: Needs some support
3: Competent - little or no support required

		Mentee Self-Assessment			Mentor Assessment		
		Confident	Needs more support	N/A	Score (1-3)	N/A	Mentor Notes / Score Explanation
	**A. Pre-ART**						
1	Records relevant patient history (TB, ARVs, OI, PMTCT, reviews lab results etc., prior notes).						
2	Completes head-to-toe physical exam (checks for vital signs, weight, height, nutritional status, pregnancy status, etc.) and treats/refers [illegible]						
3	Demonstrates ability to identify exposed infants <24 months and PSHD in infants <12 months.						

#		Confident	Needs more support	N/A	Score (1-3)	N/A	Mentor Notes / Score Explanation
4	Screening of all HIV patients for TB in each clinical visit according to the standard checklist.						
5	Prescribes CPT and IPT for adults and children appropriately.						
6	Able to interpret DNA PCR and rapid test results in children aged <12 months and >12 months and ensures HIV+ infants start ART immediately.						
7	Demonstrates ability to correctly identify *adults* eligible for ART based on WHO clinical staging, universal access or immunologic staging and [illegible]						
8	Demonstrates ability to correctly identify *children* eligible for ART based on WHO clinical staging, universal access or immunologic staging and [illegible]						
9	Implements and follows up on scheduled CD4 testing for pre-ART.						
10	Knows when to send lab tests (Hb, LFT's) and interprets results correctly (e.g. anaemia, hepatitis etc.).						
				**Totals**	a	b	

Calculate total score: (**100 x** [a]) **÷** (**30 –** [b]) **=** [ ] **%**

Based on %, tick: **[ ] RED (0-49%)** **[ ] ORANGE (50-79%)** **[ ] GREEN (80-100%)**

• *Mentee Self-Assessment Column = Completed by Mentee*<br>• *Mentor Assessment Column = Completed by Mentor*		**Mentee Self-Assessment**			**Mentor Assessment**		**<u>Mentor scoring:</u>**<br>*1: Needs intensive support*<br>*2: Needs some support*<br>*3: Competent - little or no support required*
	**B. ART**	**Confident**	**Needs more support**	**N/A**	**Score (1-3)**	**N/A**	**Mentor Notes / Score Explanation**
1	Correctly selects appropriate treatment regimen for all patients (including TB, pregnant, lactating and paediatric patients).						
2	Calculates adherence using doses missed and pill counts (using job aid if necessary).						
3	Recognises toxicity/side effects, grades the severity and manages appropriately - mentee should demonstrate capability to identify side effects [illegible]						
4	Recognises and treats IRIS appropriately.						
5	Submits and interprets viral load samples on schedule (6 months, 2 years, and every 2 years thereafter).						
6	Recognises treatment failure and responds/refers appropriately.						
7	Appropriately recognises when patients should be referred for further management.						
				**Totals:**	c	d	

Calculate total score: (**100 x** [c]) **÷** (**21 –** [d]) **=** [ ] **%**

Based on %, tick: **[ ] RED (0-49%)** **[ ] ORANGE (50-79%)** **[ ] GREEN (80-100%)**

• *Mentee Self-Assessment Column = Completed by Mentee*
• *Mentor Assessment Column = Completed by Mentor*

Mentor scoring:
1: Needs intensive support
2: Needs some support
3: Competent - little or no support required

		Mentee Self-Assessment			Mentor Assessment		
	**C. PMTCT and paediatrics**	**Confident**	**Needs more support**	**N/A**	**Score (1-3)**	**N/A**	**Mentor Notes / Score Explanation**
**1**	Provides HIV testing and counseling in pregnancy, and appropriate access to contraception at 6 weeks postnatal visit.						
**2**	Provides and educates on infant PMTCT prophylaxis (6 weeks NVP from birth) at ANC, delivery and post-natal; and CTX from age 6 weeks [illegible]						
**3**	PITC for all negative or unknown HIV status women upon admission to labour ward or new in postnatal clinic.						
**4**	Initiates all newly confirmed HIV+ pregnant women on appropriate ART regimen; women already on ART remain on same regimen as before.						
**5**	Refers HIV+ women for ongoing ART care post-delivery and ensure child is enrolled as exposed infant in parallel.						
**6**	Applies appropriate risk reduction measures to prevent transmission during labour and delivery.						
**7**	Ensures that DNA PCR testing is completed in exposed infants at 6 weeks of age followed by confirmation RT at 12m and 24m age.						
**8**	Counsels on infant feeding, breastfeeding, complementary feeding and weaning practices.						
**9**	Discharges uninfected exposed infants 6 weeks after cessation of breastfeeding (assess at 24 months).						
**10**	Ensures ART initiation is done in PSHD and universally eligible children <12m and <24m respectively.						
**11**	Able to prescribe paediatric formulation according to weight and explain to care-giver/parent paediatric dosing.						
				**Totals:**	e	f	

Calculate total score: (**100 x** [e]) **÷** (**33 –** [f]) **=** [ ] **%**

Based on %, tick: **[ ] RED (0-49%)** **[ ] ORANGE (50-79%)** **[ ] GREEN (80-100%)**

	• *Mentee Self-Assessment Column = Completed by Mentee*<br>• *Mentor Assessment Column = Completed by Mentor*	**Mentee Self-Assessment**			**Mentor Assessment**		**Mentor scoring:**<br>*1: Needs intensive support*<br>*2: Needs some support*<br>*3: Competent - little or no support required*
	**D. Additional topics**	**Confident**	**Needs more support**	**N/A**	**Score (1-3)**	**N/A**	**Mentor Notes / Score Explanation**
**1**	Provides ongoing adherence counseling and support.						
**2**	Advises patients on positive living - proper nutrition, alcohol/drug cessation, sexual risk reduction, HIV/AIDS disease progression, transmission, [illegible]						
**3**	Follows up on partner/children HIV status ascertainment.						
**4**	Treats patients with empathy, dignity and respect, using appropriate language and protects their privacy and confidentiality.						
**5**	Recognises and treats STIs and provides dual protection (contraception and condoms) to all patients.						
**6**	Provides PEP within 72 hours of sexual or occupational exposure to HIV.						
**7**	Clearly documents and updates patient health passport, mastercard and register.						
				**Totals:**	g	h	

Calculate total score: (**100 x** [g]) **÷** (**21 –** [h]) **=** [ ] **%**

Based on %, tick: **[ ] RED (0-49%)** **[ ] ORANGE (50-79%)** **[ ] GREEN (80-100%)**

Once the assessment is complete, transfer the total % scores from each section to the boxes on the front page to calculate the overall % score.

Please add additional comments below and on the back of this page

....

....

....

....
