# Supplementary material for: Successes and Challenges of HIV Mentoring in Malawi: The Mentee Perspective
Source: PLoS One. 2016 Jun 28;11(6):e0158258. doi: 10.1371/journal.pone.0158258 (PMC4924818; doi:10.1371/journal.pone.0158258)
Supplement: S2 Fig — (PDF) [file pone.0158258.s002.pdf]

| 2013 Health Center Dashboard | | | | | |
|---|---|---|---|---|---|
| Indicator | 1st QTR | 2nd QTR | 3rd QTR | 4th QTR | CUMULATIVE |
| # of newly registered ANC clients | 402 | 309 | 254 | 434 | 1399 |
| Percentage of pregnant women with known HIV status in ANC | 67% | 90% | 99% | 81% | 82% |
| Percentage of HIV positive women seen in ANC | 6% | 4% | 4% | 4% | 5% |
| Percentage of HIV+ pregnant women who received ARVs to reduce risk of MTCT of HIV in ANC | 100% | 92% | 70% | 100% | 92% |
| Percentage of women registered for delivery who had known HIV status during the pregnancy | 96% | 95% | 99% | 98% | 96% |
| # of previously untested women who were tested in Maternity | 5 | 8 | 1 | 5 | 19 |
| Percentage of previously untested women who were tested in Maternity | 42% | 62% | 100% | 100% | 61% |
| Percentage of women who registered in Maternity and were tested for HIV either in ANC or Maternity. | 97% | 98% | 100% | 100% | 99% |
| Percentage of HIV positive women (previous or new) who received ART for PMTCT | 100% | 100% | 100% | 100% | 100% |
| # of infants born to HIV (+) women | 15 | 11 | 4 | 9 | 39 |
| Percentage of exposed children born at site who are registered for EID | 80% | 45% | 0% | 0% | 44% |
| QTR cohort of active exposed infants registered (includes transfer ins) | 4 | 9 | 6 | 1 | 20 |
| Percentage of exposed infants who completed 6 weeks of NVP for PMTCT | 100% | 89% | 83% | 100% | 90% |
| Percentage of infants born to HIV+ pregnant women who were started on CPT at 6-8 weeks of life | 0% | 22% | 100% | 0% | 40% |
| Percentage of infants born to HIV+ women who were started on CPT within 3 months of birth | 0% | 22% | 100% | 0% | 40% |
| Percentage of infants born to HIV+ women who received HIV-PCR testing at 6-8 weeks of life | 75% | 0% | 0% | 0% | 15% |
| Percentage of infants born to HIV+ women who received HIV-PCR testing within 3 months of birth. | 100% | 0% | 0% | 0% | 20% |
| Total # of DBS samples sent during the cohort months regardless of the age of the infant | 3 | 9 | 5 | 7 | 24 |
| Percentage of HIV-PCR sample results received at healthcare facility withing one month from the date of collection | 67% | 0% | 0% | 14% | 13% |
| Percentage of positive HIV-PCR samples | 0% | 0% | 0% | 0% | 0% |
| Percentage of exposed infants who had any HIV test (rapid HIV or HIV-PCR) by 12 months of age (includes transfer-ins) | 71% | 73% | 100% | 75% | 75% |
| Percentage of positive HIV tests at site | 6% | 6% | 5% | 4% | 5% |
| First time initiations at site | 29 | 24 | 22 | 42 | 117 |
| Percentage of patients newly initiated on ARVs based on CD4 count versus clinical staging | 20% | 29% | 42% | 70% | 47% |
| Percentage patients who died within 3 months of starting ART | 0% | 0% | 8% | 0% | 2% |
| Percentage of adults and children known to be alive and on treatment 12 months after initiation of ART | 100% | 98% | 100% | 65% | 93% |
| Percent of HIV+ persons receiving cotrimoxazole prophylaxis | 100% | 100% | 96% | 89% | |
| Percentage of HIV+ patients who were screened for TB in HIV care or treatment settings | 100% | 100% | 78% | 91% | XXX |
| # of eligible HIV positive patients who received Isoniazid Preventative Therapy (IPT) at their last visit | 6 | 12 | 4 | 14 | 14 |
